# The effect of adolescents’ perceived social support types on sense of meaning in life: the mediating role of social- emotional competence

**DOI:** 10.3389/fpsyg.2025.1585270

**Published:** 2025-09-04

**Authors:** Chanjuan Zhang, Qiuying Wang, Wenyin Zhao, Haibo Yu, Chao Ma

**Affiliations:** ^1^Teacher’s College, Shihezi University, Shihezi, China; ^2^Center of Application of Psychological Research, Shihezi University, Shihezi, China

**Keywords:** perceived social support, social-emotional competence, sense of meaning in life, latent profile analysis, adolescents

## Abstract

**Background:**

Lacking a sense of meaning in life has been linked to negative emotions, anxiety, depression, suicidal ideation and suicide, and antisocial behavior. While perceived social support influences the sense of meaning in life, existing research has not precisely categorized perceived social support, with limited research on how a sense of meaning in life is expressed. This study aimed to examine how different types of perceived social support affect adolescents’ sense of meaning in life and the mediating role of social-emotional competence.

**Methods:**

Using the Multidimensional Scale of Perceived Social Support, the Delaware Social and Emotional Competency Scale, and the Sense of Meaning in Life Scale, data were collected from 1,145 students from four middle schools in Province H, China. Latent profile analysis using Mplus 8.0 identified differences in social-emotional competence and sense of meaning in life across different perceived social support types. The mediating role of social-emotional competence between adolescents’ perceived social support types and sense of meaning in life was also examined.

**Results:**

A follow-up analysis of the 1,145 data collected showed that: (1) Four potential categories of adolescent perceived social support existed: Poor (6.03%), Rich (25. 15%), Peer-oriented type (7. 16%), and Moderate (61.66%). Adolescents in these categories differed significantly in their social-emotional competence and sense of meaning in life, with both of the latter being stronger among adolescents in the Rich category than those in the Poor category. Taking the Poor type as the reference group, social-emotional competence mediated the relationship between the other groups and the sense of meaning in life.

**Conclusion:**

There is variation in adolescents’ perceived social support and social-emotional competence, which should be considered when designing interventions to enhance the sense of meaning in life. Educational practice should focus on enhancing adolescents’ social-emotional competence and the sense of meaning in life. Additionally, peer support groups should be nurtured, taking into account the core influence of peers on adolescents’ development, encouraging cooperation, interaction, and social interaction.

## 1 Introduction

Meaning in life refers to a psychological experience in which individuals subjectively perceive their lives as comprehensible and guided by meaningful goals. It is increasingly recognized as a protective factor in supporting adolescents’ mental health (George and Park, 2017). However, a lack of a sense of meaning in life has been linked to an increase in problematic behaviors such as social adjustment disorder, mobile phone dependence, depression, and suicidal behaviors (Kishimoto Pengzi and Shanshan, 2022). Such behaviors not only hinder individual psychological health but are also closely associated with an increased risk of criminal behaviors (Zhao et al., 2022). Previous studies have shown that suicides among adolescents aged 15–24 in China account for more than 26% of the total number of suicides (Zhang J. et al., 2022); to some extent, this worrying statistic is directly related to China’s current lack of emphasis on life education for adolescents. Therefore, understanding possible ways to enhance adolescents’ sense of meaning in life and providing targeted life education to adolescents are important topics for in-depth study within the field of mental health education.

### 1.1 Cumulative ecological resources perspective

Positive psychology suggests that adolescents’ search for meaning in life requires access to supportive resources: a set of relevant experiences, skills, and values. Based on Bronfenbrenner’s ecosystem theory, Benson divided resources into internal (e.g., mental health, resilience to face challenges) and external (e.g., parental emotional support) resources (Benson et al., 1999). Current research has mainly explored ways to enhance the sense of meaning in life from a single perspective, focusing on either external resources (such as parenting style, family capital, teacher–student relationships, peer atmosphere) or internal resources (such as emotional experiences and Big Five personality traits) (Chen Yinghua, 2016; Li, 2023; Qian and Cao, 2023; Ma, 2024). Although this research approach is useful for analyzing the role of specific resources, it tends to overlook the overall effect of the interaction between internal and external resources on the development of social-emotional abilities.

Cumulative ecological resources theory builds on positive psychology and ecological systems theory to propose the concept of “ecological resources,” moving beyond the traditional dichotomy of internal and external resources. This theory asserts that teenagers’ real-life environment contains both internal and external resources. Within this environment, they acquire ecological resources through interactions with multiple social systems, including their family, school, and neighborhood. The basic theory derives from the notion that effective positive development is related to individuals’ accumulation of ecological resources. Multiple empirical studies have verified the positive impact of accumulated ecological resources on adolescents’ sense of meaning in life. Such resources have been shown to effectively alleviate depressive symptoms, reduce the incidence of suicidal and self-harming behaviors, and play a positive role in shaping adolescents’ core values and social-emotional abilities. However, the role of perceived social support—a key internal and external element of the ecosystem of young people—in promoting a sense of meaning in life has not yet been systematically and comprehensively studied.

### 1.2 Understanding the heterogeneity of social support

Perceived social support refers to individuals’ subjective perception of support from others, such as parents and friends. It can help them cope with stressful events and release negative emotions, thus promoting their physical and mental health (Brissette et al., 2002). Within the framework of accumulated ecological resources, differences in how young people understand the three types of social support resources (family, peers, and teachers) are due to particular psychological mechanisms. Young people perceive ecological resources in different ways; combined with the diversity and complexity of these resources, this means that their understanding of social support in real life is often heterogeneous (Guo et al., 2021; Wang et al., 2023).

Previous studies of perceived social support among adolescents have largely taken a variable-centered approach (Chen, 2022). However, this approach has limitations in revealing the heterogeneity of perceived social support, leaving unanswered several key questions: How can adolescents’ perceived social support be precisely categorized? And what is the impact of perceived social support on sense of meaning in life when the internal heterogeneity of perceived social support is taken into account? According to developmental contextualism, individual growth is clearly non-linear and there is no fixed developmental path (Steger et al., 2008). A growing body of research has confirmed that adolescents show significant individual differences in their perceptions of social support, which can be classified as high, moderate, and low levels (Steger et al., 2008; Liu et al., 2011; Guo et al., 2021; Chen, 2022; Zhang, 2022; Wang et al., 2023). The individual-centered approach of latent profile analysis (LPA) can effectively identify heterogeneity among individuals, and international scholars have used it to classify adolescents’ perceived social support into high, medium, and low categories (Petersen et al., 2023). Certain scholars have categorized adolescents’ perceived family support into low and high groups, finding that adolescents with higher perceived family support were less likely to experience depression during the developmental process (Fang et al., 2020). However, in China, research on classifying types of perceived social support among adolescents is still in its infancy. Therefore, it is necessary to examine the different categories of social support perceived by adolescents through an individual-centered analytical approach.

Given that adolescents perceive varying levels of support from family, peers, and teachers, the combination of these support resources may be more than merely quantitatively cumulative, resulting in distinct patterns with unique characteristics. Therefore, this study adopts a person-centered research perspective and puts forward Research Question 1: Are there several potential categories of perceived social support in the adolescent population? Based on this, the first research hypothesis was formulated:

Hypothesis 1: Adolescents’ perceived social support can be categorized into a number of potential categories with distinct characteristics.

### 1.3 Impact of perceived social support on sense of meaning in life

The theory of sense of meaning in life posits that good interpersonal relationships and a strong social support system are important for individuals to gain a sense of meaning in life. During development, adolescents actively engage in cognitive processing, in which they notice, perceive, interpret, and evaluate external support resources. Through such cognitive processing, they come to perceive the social support available to them. Perceived social support involves not only the psychological qualities of individual subjective well-being and self-confidence but also a wide range of external interpersonal resources, including perceived family support, perceived peer support, and perceived teacher support. In the family context, parental care and parent–child relationship intimacy have been shown to have a positive effect on reducing adolescents’ suicidal ideation (Pei et al., 2024), while adolescents’ sense of meaning in life was found to be stronger when they perceived a democratic family atmosphere (Yaoting et al., 2024). Adolescents’ perceived teacher support promotes the development of school connectedness, which in turn enhances their sense of meaning in life (Liangliang, 2023). At the peer level, adolescence is a critical period for developing self-identity. Feeling accepted, understood, and emotionally supported by peers can satisfy adolescents’ strong need for a sense of belonging, help them establish their value within the collective, effectively buffer academic pressure and developmental challenges, and thus enhance their perception of the value and meaning of life (Lin, 2023).

The above studies clearly reveal that support from the three key developmental contexts of the family, school, and peers plays an indispensable role in constructing adolescents’ sense of meaning in life. On this basis, ecological resource theory further proposes that perceived developmental resources such as family support, peer support, and teacher support not only work individually but also cumulatively, and that the cumulative effect of ecological resources in multiple contexts is more strongly associated with positive developmental outcomes for adolescents than ecological resources in a single context (Benson et al., 2006). For example, a previous study of 2,710 left-behind children found an exponential increase in the impact of cumulative family and school developmental resources on psychological resilience (Yang et al., 2023). A follow-up study of adolescents in Yunnan showed that individuals with only single-context high support (e.g., family support or teacher support) had significantly lower academic self-concept scores than those with high support from two contexts (Juan, 2023). Based on the above discussion, the present study proposes Research Question 2 based on Hypothesis 1: Do distinct categories of perceived social support differentially predict adolescents’ level of sense of meaning in life? To this end, the second hypothesis is proposed: there is a significant difference in the level of sense of meaning in life across adolescents’ perceived social support categories.

Hypothesis 2a: The “overall high support” category has the highest meaning of life scores.

Hypothesis 2b: The “overall low support” category has the lowest meaning of life scores.

Hypothesis 2c: There is a gradient in the differences in the mean level of meaning of life between the categories, following the order: “overall high support”>“partial support”>“overall low support.”

### 1.4 Impact of social support on social-emotional competence

Social support theory suggests that individuals’ perceived social support contributes to their psychological resilience and social adaptability (Li et al., 2023). Social-emotional competence, as a core competency in the development of social adaptive capacity, can be defined as individuals’ ability to perceive and regulate their own and others’ emotions in social interactions. It can be categorized into four dimensions: social awareness, self-management, peer relations, and responsible decision-making (Huang, 2022). Adler’s theory of sense of community states that an individual’s ability to “accept the self,” “trust the other,” and “contribute to the other” is the core of social support. The cumulative development of resources, which is also emphasized in bioecological theory, provides an integrative framework for understanding how social-emotional competence develops (Bronfenbrenner and Morris, 2006). The theory emphasizes that an individual’s development occurs primarily through ongoing interaction with people, objects, and symbols in their immediate environment. Rather than absorbing a single resource in isolation, adolescents cumulatively access and integrate internal and external resources through daily interactions with key others in their nested microsystems of family, school, and peer groups. This cross-situational ecological resource provides a critical developmental foundation for shaping social-emotional competence through the cumulative experience of close interactions, especially when combining diverse patterns of support from different key developmental agents.

Empirical studies have also shown that social-emotional competence is significantly and positively correlated with individuals’ perceived social support (Adler, 1966), and scholars have found that perceived social support has a positive predictive effect on social-emotional competence in systematic psychometric measurements of groups of high school students (Deci and Ryan, 2000). Individuals’ different combinations of perceived social support have been found to be significantly associated with their levels and characteristics of social-emotional competence (Dirik and Göcek-Yorulmaz, 2018). In summary, within the cumulative ecological resources perspective, drawing on different combinations of types of social support is a key factor in enhancing adolescents’ social-emotional competence (Sun, 2023). Based on the above discussion, Research Question 3 is proposed on the basis of the first hypothesis: Do different potential categories of perceived social support differentially predict adolescents’ social-emotional competence? To this end, the third research hypothesis is proposed: there is a significant difference in social-emotional competence across different categories of perceived social support.

Hypothesis 3a: Adolescents in the “overall high support” category have the highest social-emotional competence scores.

Hypothesis 3b: Adolescents in the “overall low support” category have the lowest social-emotional competence scores.

Hypothesis 3c: There is a gradient in the differences in the mean level of social-emotional competence between the categories, following the order: “overall high support”>“partial support”>“overall low support.”

### 1.5 The mediating role of social-emotional competence

According to the conservation perspective of cumulative ecological resources, individuals strive to acquire and maintain the resources they value to facilitate adaptation, growth, and goal attainment (Halbesleben et al., 2014). Family, peer, and teacher support are key social resources in the ecosystem of adolescent development. These resources are not static, and their effectiveness depends on how they are perceived, internalized, and transformed by the individual. Social-emotional competence—as a core predictor of future life satisfaction, well-being, and sense of meaning in life (Tian et al., 2021; Ke et al., 2022)—is the key competence capital that is formed when individuals internalize and transform ecological resources. The model of constructing a sense of meaning in life posits that this process involves comparing, assessing, and judging the stimuli obtained from a situation in light of existing beliefs. Individuals with higher social-emotional competence are more capable of integrating emotional information. They can obtain a new sense of meaning by assimilating and moderating the meaning of a particular event, resulting in a stronger sense of meaning in life (Zhao et al., 2017).

Moreover, the social-emotional competence school model highlights that developing social-emotional competence motives and behaviors within the school environment promotes the fulfillment of basic psychological needs (Collie, 2019). This, in turn, gradually enhances individuals’ level of psychological resilience and self-efficacy, thus contributing to a deeper sense of meaning in life (Deing et al., 2021). The synergy of resource integration emphasized by the cumulative ecological resources theory is particularly relevant here. When individuals perceive rich, supportive resources across contexts, their social-emotional abilities are more likely to be stimulated, thus generating a gainful effect that extends beyond the impact of a single resource. This cumulative effect provides a stronger impetus for the deeper development of a sense of meaning in life. Based on the above discussion, Research Question 3 is posed: What role does social-emotional competence play between understanding different perceived categories of social support and a sense of meaning in life? To this end, the fourth research hypothesis is proposed:

Hypothesis 4: Social-emotional competence mediates between different perceived categories of social support and a sense of meaning in life.

In summary, based on the perspective of cumulative ecological resources, this study aimed to examine how adolescents’ perceived social support impacts their sense of meaning in life, with social-emotional competence playing a mediating role. The specific research objectives were to (1) identify different categories of perceived social support among adolescents (Sumin and Junya, 2024); (2) examine how the characteristics of different categories of perceived social support affect adolescents’ sense of meaning in life; (3) examine how the characteristics of different categories of perceived social support affect social-emotional competence; (4) explore the mediating role of social-emotional competence in the relationship between categories of adolescents’ perceived social support categories and their social-emotional competence.

## 2 Materials and methods

### 2.1 Participants and procedure

Using SPSS 26.0 software for sample size estimation, and with reference to data from previous studies, the overall standard deviation σ of perceived social support was calculated as 10.79. The confidence level was set as 0.95, the significance level α at 0.05, and the allowable error δ at 0.8. Using the sample size calculation formula *n* = (μασ/δ)2, and taking into account instances of no response or invalid responses, the response rate was set to 90%, while the minimum sample size was calculated to be 776. To account for the possibility of invalid responses, missing data, or other unforeseen circumstances, the sample size was set to be between 300 and 1,000 to improve model robustness and the reliability of results.

Using school classes as the sampling unit, and with the consent of the schools and students, 1,185 questionnaires were distributed in four middle schools in Shanxi Province between October and November 2023 using convenience sampling. After removing incomplete answers and unreliable questionnaires, 1,145 valid questionnaires were obtained, yielding a recovery validity rate of 96.62%.

### 2.2 Measures

#### 2.2.1 The multidimensional scale of perceived social support

This scale was compiled by Zimet et al. (1988) and revised by Yan and Zheng (2006). It consists of three sub-scales: Perceived Family Support, Perceived Peer Support, and Perceived Teacher Support. Each sub-scale contains four items measured with a seven-point scoring method: the higher the total score of the three sub-scales, the better the degree of perceived social support of the individual. The Cronbach’s alpha coefficient for the aggregate scale in this study was 0.89, and the Cronbach’s α coefficients of the three sub-scales of Perceived Family Support, Perceived Peer Support, and Perceived Teacher Support were 0.87, 0.87, and 0.91, respectively, which indicates that the scale has good reliability and validity.

#### 2.2.2 Delaware social and emotional competency scale (DSECS)

The scale was compiled by Mantz et al. (2018) and is divided into four dimensions: social awareness, self-management, peer relationships, and responsible decision-making (Song and Zhou, 2021). The scoring method is based on a four-point Likert scale, The Cronbach’s alpha coefficient for this scale in this study was 0.70.

#### 2.2.3 Sense of meaning in life scale

This scale was compiled by Wang Xinqiang and Zhang (2016) and is divided into two dimensions: having a sense of meaning in life and seeking a sense of meaning in life. The scoring method is based on a seven-point Likert scale, and the second question is reverse scored. Higher total scores indicate a stronger sense of meaning in life. The Cronbach’s alpha coefficient for the scale in this study was 0.85.

### 2.3 Research procedures

Data collection was carried out in a group practical test, using paper-and-pencil questionnaires and with consistent instructions. The staff involved in the testing were either psychology students or the researcher herself.

Before the formal survey, the researcher introduced the purpose and process of the study to school leaders and teachers, sought permission to conduct the research, and distributed informed consent forms to the subjects. At the time of the survey, the subjects were again informed in detail about the purpose and process of the study, and all the participants signed informed consent forms in a fully informed and voluntary manner.

### 2.4 Analysis strategy

In the first step of the analysis, data were initially organized and analyzed using SPSS 26.0, followed by a LPA of perceived social support using Mplus 8.3. The three dimensions of adolescents’ perceived social support were used as indicators to establish a potential profile model. One to five categories were set up to estimate the potential profile model fit and to observe the changes in the model’s fit indicators. The following indicators were selected as the criteria for choosing the number of profiles (Peugh and Fan, 2013): (1) relative fit index, including AIC, BIC, and aBIC—the lower the value, the higher the degree of fit of the model; (2) entropy—the closer the value of entropy is to 1, the more accurate the classification; (3) LMRT and BLRT indicate the difference between two neighboring models—if the results of LMRT and BLRT are significant then it means that the K model is better than the K- 1 model; (4) the category probability of each subgroup of no less than 5% (Spurk et al., 2020).

In the second step, based on the results of the latent profile analyses, a one-way ANOVA was used to test for significant differences in individual scores on sense of meaning in life and social-emotional competence in the context of different latent types of adolescents’ perceived social support, respectively. F-tests were used to determine whether the overall differences were significant or not, and post hoc tests were conducted in the case of significance (e.g., Tukey’s HSD) to determine the specific groups of differences. Means and standard deviations for sense of meaning of life and social-emotional competence were calculated for each group to characterize specific patterns of difference. In the third step, the SPSS 26.0 PROCESS macro was used to test the mediating role of social-emotional-competence between adolescents’ perceptions of the type of social support and their sense of meaning in life.

## 3 Results

### 3.1 Common method bias test

In this study, all the measures of perceived social support, sense of meaning in life, and social-emotional competence were subjected to unrotated exploratory factor analysis. Seven common factors with an eigenroot greater than 1 were extracted. The first factor explained 25.04% of the variance, which is less than the 40% critical value, indicating no significant common methodological bias in this study.

### 3.2 Descriptive statistics and correlation analysis

Table 1 provides demographic information on the survey sample, of which 551 were male and 594 were female; 240 were aged 12–13 years, 500 were aged 1–15 years, 2,394 were aged 16–17 years, and 8 were aged 18–19 years; 646 had their family residence in towns and cities, and 497 had their family residence in rural areas.

**TABLE 1:** Basic statistics of the sample (*n* = 1,145).

Categories		Number of people	Percentage (%)
Gender	Male	551	48.1%
	Female	594	51.9%
Age	12–13	240	21.0%
	14–15	500	43.7%
	16–17	394	34.4%
	18–19	8	0.7%
Family origin	Municipalities	646	56.4%
	Countryside	497	43.4%

Table 2 presents the results of the Pearson’s correlation analysis. The analysis found a significant positive correlation between the three variables of perceived social support, social-emotional competence, and sense of meaning in life. Furthermore, gender and parental marital status were significantly negatively correlated with perceived social support; gender and parental marital status were significantly negatively correlated with sense of meaning in life; age was positively correlated with social-emotional competence; and family origin was negatively correlated with social-emotional competence and sense of meaning in life. Therefore, gender, age, family origin, and parental marital status were used as control variables in the subsequent analysis.

**TABLE 2:** Results of descriptive statistics and correlation analysis (*n* = 1,145).

	M	SD	1	2	3	4	5	6	7
1. Gender	–	–	1	–	–	–	–	–	–
2. Family origin	–	–	0.01	1	–	–	–	–	–
3. Parents’ marital status	–	–	0.02	0.13**	1	–	–	–	–
4. Age	14.76	1.44	0.04	−0.24**	−0.15**	1	–	–	–
5. Perceived social support	4.93	1.05	−0.07*	−0.05	−0.11**	0.00***	1	–	–
6. Social-emotional competence	3.18	0.49	0.01	−0.10**	−0.05	0.11**	0.40**	1	–
7. Sense of meaning in life	5.04	1.05	−0.07*	−0.07*	−0.09**	0.03	0.51**	0.38**	1

*p < 0.05,

**p < 0.01,

***p < 0.001; the same applies below.

### 3.3 Potential profiling of perceived social support for adolescents

#### 3.3.1 Determination of optimal model

Table 3 shows the results of the potential profile analysis. The analysis found that as the number of categories increased, the AIC, BIC, and aBIC values gradually decreased, with the rate of decrease gradually slowing after the four-category model, indicating improved model fit. The entropy value also gradually increased with more categories. The model with four categories had an entropy value of 0.85, and the probability of each subgroup was greater than 5%. However, the LMRT *p*-value at four categories of division was not significant, while the BLRT *p*-value was significant; BLRT is a more accurate indicator of fit when both LMRT and BLRT are contradictory (Nylund et al., 2008). Thus, combining the above indicators, the four-category model was determined to be the best fit for measuring adolescents’ perceived social support.

**TABLE 3:** Fitting information for potential profile analysis of different models of adolescents’ perceived social support (*n* = 1,145).

Model	*AIC*	*BIC*	*aBIC*	*Entropy*	*LMR (p)*	*BLRT (p)*	Categorical probability
1	11,851.63	11,881.89	11,862.84	–	–	–	1.00
2	11,449.29	11,499.72	11,467.96	0.65	< 0.001	< 0.001	0.32/ 0.68
3	11,338.51	11,409.11	11,364.64	0.70	0.04	< 0.001	0.09/ 0.24/ 0.67
4	**11,248.68**	**11,339.46**	**11,282.28**	**0.85**	**0.11**	**< 0.001**	**0.06/0.62/0.25/0.07**
5	11,195.28	11,306.23	11,236.35	0.87	0.20	< 0.001	0.08/0.06/0.60/0.01/ 0.24

Lower AIC, BIC, and aBIC values indicate better model fit; higher entropy values (closer to 1) reflect clearer classification. LMR-LRT and BLRT *p*-values should be less than 0.001, with BLRT taking precedence in case of conflict. The smallest class proportion should exceed 5%. The best-fitting model is bolded.

As shown in Table 4, the average probability of belonging to each category of adolescents ranged from 85% to 94%, which meant that the results of the four-category model could be deemed credible.

**TABLE 4:** Potential category attribution probability (*n* = 1,145).

Categories	Number of subjects	Percentage	Probability of attribution			
			C1	C2	C3	C4
Class 1	69	6.03%	0.85	0.04	0.00	0.11
Class 2	706	61.66%	0.01	0.94	0.04	0.02
Class 3	288	25.15%	0.00	0.08	0.92	0.00
Class 4	82	7.16%	0.10	0.09	0.00	0.81

C1, poor type; C2, medium type; C3, rich type; C4, peer-oriented type.

**FIGURE 1:**
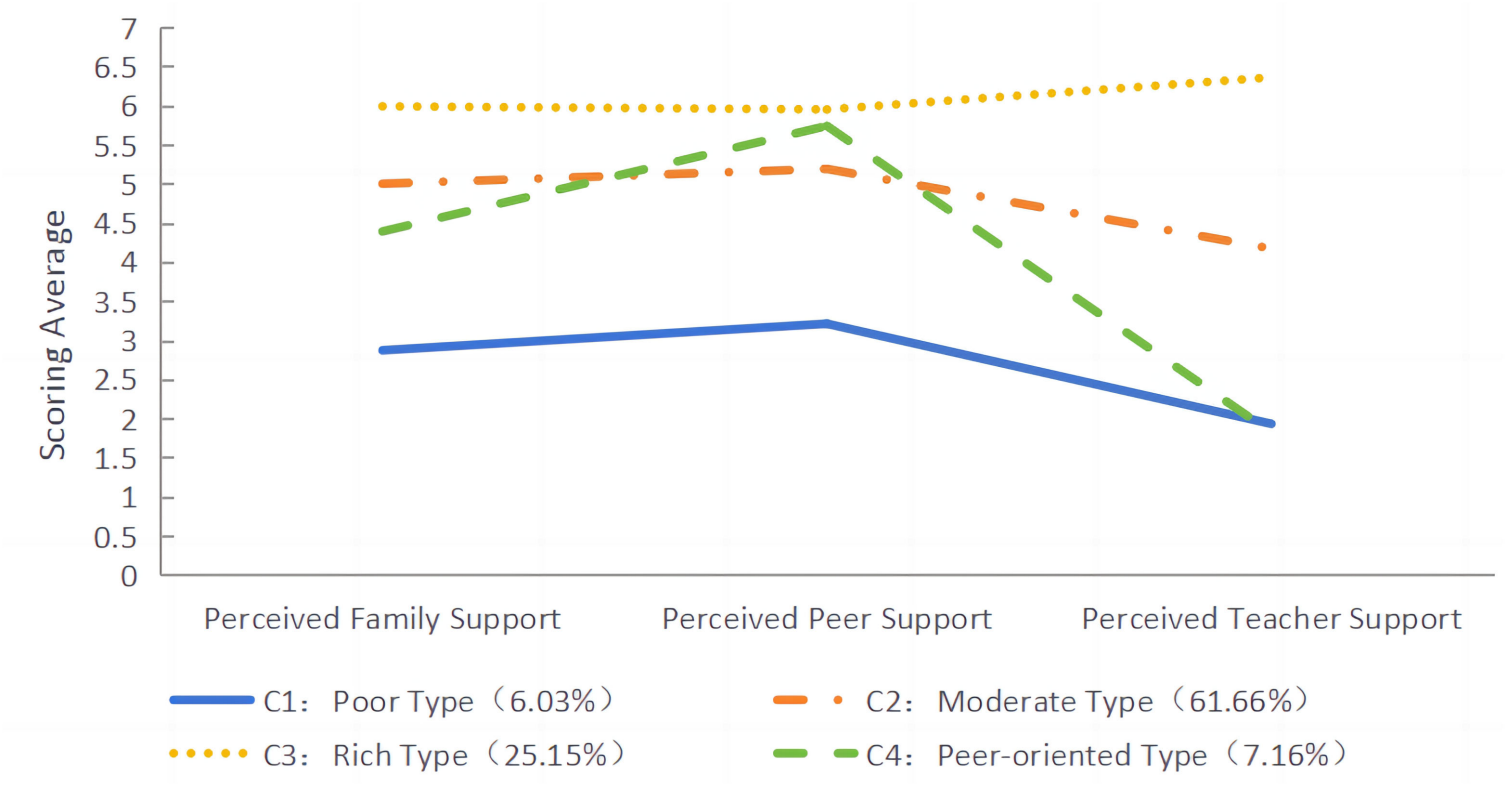
Mean scores for the four latent categories of adolescents’ perceived social support.

As shown in Figure 1, four categories of adolescents’ perceived social support were identified. Category C1 contained 6.03% of participants, who had low scores on all dimensions of perceived social support, so the category was labeled “Poor type.” Category C2 contained 25.15% of participants, whose perceived social support was at a high level, so the category was labeled “Rich type.” Category C3 contained 7.16% of participants, who perceived a high level of peer support but rarely perceived teacher support, so the category was labeled “Peer-oriented type.” Finally, Category C4 contained 61.66% of participants and was characterized by higher scores than the “Poor type” but significantly lower scores than the “Rich type” across all dimensions, so it was labeled “Medium type.”

#### 3.3 2 Differences in dimensions of perceived social support among different categories of adolescents

To determine whether the categorization of potential profiles of adolescents’ perceived social support was heterogeneous, a one-way ANOVA was conducted with perceived social support as the independent variable and perceived family support, perceived peer support, and perceived teacher support as the dependent variables. The results, shown in Table 5, indicated that the scores of the four categories of adolescents differed significantly for perceived family support (F = 150.10, *p* < 0.001, η^2^ = 0.28), perceived peer support (F = 139.42, *p* < 0.001, η^2^ = 0.27), and perceived teacher support (F = 1782.98, *p* < 0.001, η^2^ = 0.82). Further multiple comparisons showed that most of the indices had a two-by-two significant difference, which indicated the effectiveness of the potential categorization.

**TABLE 5:** Differences in total scores on perceived social support and on each dimension among different types of adolescents (M ± SD).

Items	Potential profiles of adolescents’ perceived social support				F-test	$\eta_{p}^{2}$	After-test
	C1 (69)	C2 (706)	C3 (288)	C4 (82)			
Perceived family support	2.87 ± 1.21	5.00 ± 1.15	5.99 ± 1.11	4.39 ± 1.48	150.10***	0.28	C3 > C2 > C4 > C1
Perceived peer support	3.21 ± 1.07	5.19 ± 1.04	5.95 ± 1.02	5.74 ± 0.94	139.42***	0.27	C4 > C2 > C1, C3 > C1, C3 > C2
Perceived teacher support	1.93 ± 0.73	4.17 ± 0.64	6.36 ± 0.59	1.82 ± 0.55	1,782.98***	0.82	C3 > C2 > C1, C3 > C4, C4 < C2

C1, poor type; c2, medium type; c3, rich type; c4, peer-oriented type.

*p < 0.05,

**p < 0.01,

***p < 0.001; the same applies below.

#### 3.3.3 Differences in the sense of meaning in life and social-emotional competence among adolescents with different levels of perceived social support

In order to explore the relationship between adolescents’ potential categories of perceived social support and their levels of social-emotional competence and sense of meaning in life, a one-way ANOVA was conducted. The four categories of perceived social support were the independent variables and social-emotional competence and sense of meaning in life and their corresponding dimensions were the dependent variables. The results, presented in Table 6, showed that adolescents’ types of perceived social support had a significant influence on social-emotional competence (*F* = 43.64, *p* < 0.001, η^2^ = 0.10), responsible decision-making (*F* = 25.15, *p* < 0.001, η^2^ = 0.10), social awareness (*F* = 12.30, *p* < 0.001, η^2^ = 0.03), self-management (*F* = 22.28, *p* < 0.001, η^2^ = 0.06), peer relationships (*F* = 30.59, *p* < 0.001, *η^2^* = 0.07), a sense of meaning in life (*F* = 77.27, *p* < 0.001, η^2^ = 0.17), having a sense of meaning (*F* = 84.59, *p* < 0.001, η^2^ = 0.18), and seeking a sense of meaning (*F* = 72.79, *p* < 0.001, η^2^ = 0.16). Further multiple comparisons indicated that the C2 group (Rich type) scored higher than the remaining three groups in social-emotional competence and sense of meaning in life (and their corresponding dimensions). The C1 group (Poor type) scored lower than the remaining three groups in social-emotional competence and sense of meaning of life (and their corresponding dimensions). There were significant differences between the two groups in most of the indicators.

**TABLE 6:** Descriptive data and test of the difference between the four groups of subjects on social-emotional competence and sense of meaning in life (M ± SD).

Items	Sense of meaning in life	Presence of meaning	Search for meaning	Social-Emotional competence	Responsible decision-making	Social awareness	Self-management	Peer relationships
C1	4.15 ± 1.19	3.54 ± 1.58	4.09 ± 1.29	2.85 ± 0.53	2.92 ± 0.61	2.96 ± 0.70	2.72 ± 0.58	2.79 ± 1.05
C2	4.88 ± 0.93	4.60 ± 1.22	4.79 ± 0.98	3.12 ± 0.43	3.15 ± 0.51	3.17 ± 0.59	3.00 ± 0.77	3.16 ± 0.55
C3	5.73 ± 0.94	5.71 ± 1.13	5.66 ± 0.97	3.43 ± 0.53	3.50 ± 0.95	3.43 ± 0.97	3.34 ± 0.57	3.46 ± 0.54
C4	4.81 ± 1.10	4.39 ± 1.47	4.63 ± 1.13	3.12 ± 0.49	3.17 ± 0.66	3.12 ± 0.74	2.96 ± 0.69	3.22 ± 0.59
F	77.27***	84.59***	72.79***	43.64***	25.15***	12.30***	22.28***	30.59***
$\eta_{\text{p}}^{2}$	0.17	0.18	0.16	0.10	0.06	0.03	0.06	0.07
After-test	C3 > C4 > C1, C3 > C2, C2 > C1	C3 > C4 > C1, C3 > C2, C2 > C1	C3 > C4 > C1, C3 > C2, C2 > C1	C3 > C4 > C1, C3 > C2, C2 > C1	C3 > C4 > C1, C3 > C2, C2 > C1	C3 > C2 > C1, C3 > C4	C3 > C4 > C1, C3 > C2, C2 > C1	C3 > C4 > C1, C3 > C2, C2 > C1
C1 vs. C2	−0.72*	−1.06*	−0.71*	−0.27*	−0.23*	−0.22*	−0.29*	−0.36*
C1 vs. C3	−1.58*	−2.17*	−1.57*	−0.58*	−0.59*	−0.47*	−0.62*	−0.67*
C1 vs. C4	−0.65*	−0.85*	−0.54*	−0.26*	−0.25*	−0.16	−0.24*	−0.43*
C2 vs. C3	0.85*	1.11*	0.87*	0.31*	0.36*	0.25*	0.33*	0.31*
C2 vs. C4	−0.07	−0.21	−0.17	−0.00	0.02	−0.06	−0.04	0.06
C3 vs. C4	0.92*	1.33*	1.03*	0.31*	0.34*	0.31*	0.38*	0.24*

*p < 0.05,

**p < 0.01,

***p < 0.001.

### 3.4 Mediation effect test

**FIGURE 2:**
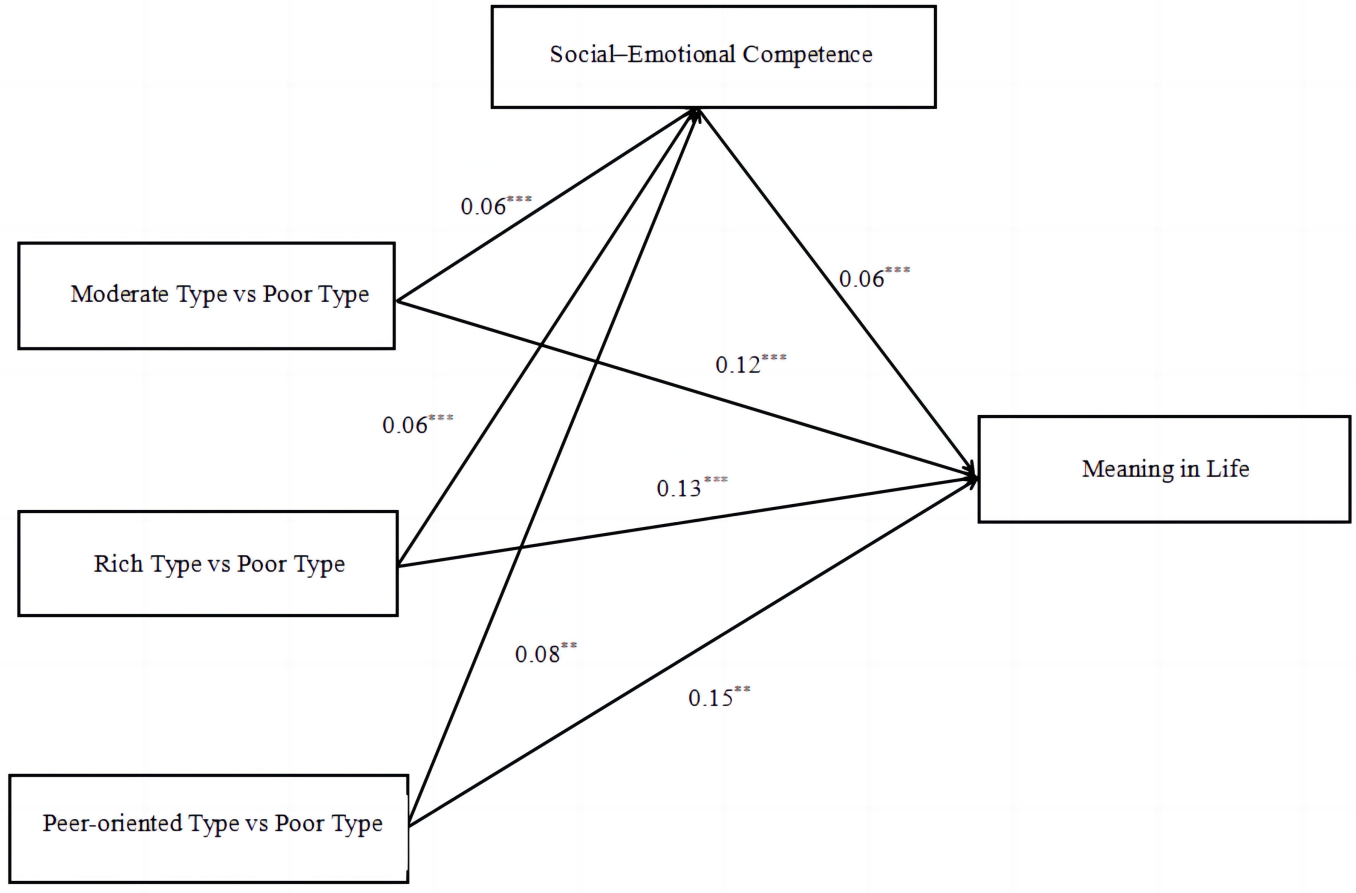
Mediation model of the effects of adolescents’ perceived social support type and social-emotional competence on their sense of meaning in life. *p < 0.05, **p < 0.01, ***p < 0.001.

Mediation effect analysis was conducted using the PROCESS macro in SPSS, with Model 4 selected. Gender, age, family origin, and parental marital status were used as covariates. Adolescents’ type of perceived social support was set as the categorical independent variable, with dummy coding used to assign the “Poor type” group as the reference group. Adolescents’ sense of the meaning of life was set as a dependent variable, while adolescents’ social-emotional competence was set as the mediating variable. The Bootstrap method was used with 5,000 resamples. As shown in the model diagram (Figure 2), adolescents’ type of perceived social support affected their sense of the meaning in life through two paths—direct path: adolescents’ type of perceived social support → sense of meaning in life; indirect path: adolescents’ type of perceived social support → social-emotional competence → sense of meaning in life.

As shown in Table 7, the direct path effect values of the Rich, Peer-oriented, and Medium types of perceived social support on sense of meaning in life relative to the Poor type were 1.19, 0.44, and 0.51, respectively (95% CI: 0.93∼1.45; 95% CI: 0.14∼0.74; 95% CI: 0.27∼0.74). The indirect path effect values were 0.34, 0.14, 0.15 (95% CI: 0.23∼0.46; 95% CI: 0.04∼0.25; 95% CI: 0.06∼0.24), with the Rich type showing the largest indirect path effect as follows: Rich type → social-emotional competence → sense of meaning in life.

**TABLE 7:** Standardized effect values and 95% confidence intervals for each pathway.

Pathway	Standardized effect values	95% confidence intervals	
		Lower	Upper
Total effect of medium type	0.65	0.41	0.90
Medium type → sense of meaning in life	0.51	0.27	0.74
Medium type → social-emotional competence → sense of meaning in life	0.15	0.06	0.24
The total effect of rich type	1.53	1.27	1.79
Rich type → sense of meaning in life	1.19	0.93	1.45
Rich type → social-emotional competence → sense of meaning in life	0.34	0.23	0.46
Total effect of peer-oriented type	0.58	0.27	0.89
Peer-oriented type → sense of meaning in life	0.44	0.14	0.74
Peer-oriented type → social-emotional competence → sense of meaning in life	0.14	0.04	0.25

Using the Poor type as the reference group, the 95% CI intervals for Poor, Peer-oriented, and Medium types of perceived social support as mediators did not include 0. In comparison to the Poor type, the Rich, Peer-oriented, and Medium types all significantly and positively predicted adolescents’ social-emotional competence (β = 0.06, *p* < 0.001; β = 0.08, *p* < 0.01; β = 0.06, *p* < 0.001), while the Rich, Peer-oriented, and Medium types all significantly and positively predicted adolescents’ sense of meaning in life (β = 0.13, *p* < 0.001; β = 0.15, *p* < 0.01; β = 0. 12, *p* < 0.001). In addition, adolescent social-emotional competence also significantly positively predicted adolescents’ sense of meaning in life (β = 0.06, *p* < 0.001). Thus, the mediating effect of social-emotional competence on the sense of meaning in life was significant for the remaining three groups relative to the Poor type. That is, adolescents’ social-emotional competence played a significant role in the relationship between the Rich, Peer-oriented, and Medium types of perceived social support and the sense of meaning in life.

## 4 Discussion

### 4.1 Potential profiling results of adolescent perceived social support

In this study, potential profile analysis was used to examine adolescents’ perceived social support based on cumulative ecological resources theory. The results identified four types of social support: Poor (6.03%), Rich (25.15%), Medium (61.66%), and Peer-oriented (7.16%), thus validating Hypothesis 1. Based on cumulative ecological resource theory, this pattern of differentiated combinations of social support reflects the heterogeneity of how adolescents acquire and integrate resources within nested ecosystems such as the family, school, and among peers. Previous studies have used structural magnetic resonance imaging, behavioral cognitive tests, and gene/methylation histology data from a longitudinal cohort of adolescents to reveal heterogeneity in the structural developmental patterns of whole-brain gray matter volume (GMV) in adolescent populations. These developmental patterns have been found to significantly influence an individual’s ability to process and utilize environmental resources (Shi et al., 2024). This finding provides a strong neurobiological basis for the theory of cumulative ecological resources, whereby differences in the physiological development of different individuals directly affect their ability to perceive the ecological resources around them, which in turn leads to the emergence of different categories of perceived social support.

Moreover, the classification of perceived social support in this study is similar to previous studies’ classification of Low Relational Support, Moderate Relational Support, Peer-Oriented Support, and High Relational Support. In terms of distribution ratios, adolescents’ perceived social support generally showed high levels, which is consistent with the findings of previous studies (Nylund et al., 2008). However, this study found some differences in the proportion of adolescents categorized under the Peer-oriented type of perceived social support compared to studies conducted in other countries. For example, in another study 13.2% of adolescents were categorized into the Peer-Oriented Support group, compared to 23% categorized into the Peer-oriented group in the current study (Shin et al., 2021). From the perspective of cumulative ecological resources theory and taking a cross-cultural view, this difference may reflect the role of China’s unique collectivist cultural context in shaping ecological resources, especially in perceiving teacher support. First, the Chinese cultural context emphasizes collectivism, which leads to individuals being more likely to seek peer group identification during their development, thus weakening their reliance on teacher support. Second, the Chinese education system tends to emphasize authority and discipline, with teachers often playing an authoritative role, which influences students’ perceptions of teacher support. Third, collectivist families rely on implicit associations, avoiding directly seeking help in order to maintain relational harmony. In contrast, individualistic families tend to externally instrumentalize help-seeking, view support as a quantifiable resource, and value the timeliness and relevance of the response. Finally, a lack of cooperation between home and school makes peer support a compensatory channel for the lack of teacher support, which is a phenomenon that is particularly salient in the high-pressure environment of East Asian education (Yan, 2024). However, this may be only one of the reasons behind the above finding, and further study is needed to identify whether there are other influencing factors.

The core difference between the Medium and Rich types of perceived social support is the degree of ecological resource accumulation (Benson et al., 1999). Adolescents within the Rich type reach high levels of family, peer, and teacher support, creating a resource stacking effect that corroborates ecological resource theory. The synergy of multiple contextual resources is a better predictor of positive developmental outcomes than a single contextual resource (Theokas and Lerner, 2006). It is important to note in educational practice that although the Medium type accounts for the highest percentage (61.66%) of adolescents, there is still much room for improvement in terms of teacher support and self-management.

Of particular concern are the Poor and Peer-oriented types, whose formation reflect an imbalance in the accumulation of ecological resources. The former lacks a cross-system supply of resources, while the latter are forced to rely on a single system of resources to compensate for a blockage in the transmission of micro-environmental resources. Adolescents with a Poor type of perceived social support tend to have a degree of detachment from social relationships and a lack of social skills. Fortunately, this category accounted for the smallest percentage of adolescents in the study (9%). It is important for teachers to pay special attention to the adolescents with a Poor type of perceived social support and to implement interventions to help them perceive higher levels of social support.

For adolescents with a Peer-oriented type of perceived social support, significant other theory suggests that their social networks gradually changes from parent–child and teacher–student attachment to peer attachment (Xiang et al., 2024). At the same time, as adolescents grow older, their self-awareness increases, which may be accompanied by a corresponding decrease in their need for guidance from teachers. This shift in the pattern of teacher–student interactions, where teachers may tend to be more lenient or guiding rather than directive, can lead to a decrease in adolescents’ perceptions of teacher support. However, previous research has indicated that close interactions with teachers helps students to better establish good relationships with their peers by providing important information that their peers can observe (Hughes et al., 2001). Therefore, future research should prioritize exploring the mechanisms underlying the development of perceived social support in peer-oriented adolescents, while educational practice should focus on developing and evaluating intervention strategies to improve peer-supportive adolescents’ perceived social support.

### 4.2 Differences in the sense of meaning in life among adolescents with different perceived social support types

Significant differences in adolescents’ sense of meaning in life across the perceived social support categories validated Hypothesis 2. Specifically, adolescents with a rich level of perceived social support demonstrated the highest level of sense of meaning in life (confirming Hypothesis 2a), while adolescents with a poor level of perceived social support showed the lowest level of sense of meaning in life (confirming Hypothesis 2b). These results corroborate the core insights of cumulative ecological resources theory. Adolescents with a rich level of perceived social support perceive and receive high-quality, diverse, and stable support resources from internal and external sources. These resources not only provide direct emotional comfort and instrumental help but, more importantly, promote the development of a sense of belonging, self-worth, and mobility, leading to a higher sense of meaning in life (Sun et al., 2011; Jiang et al., 2023). When individuals find it difficult to obtain emotional support from those around them to alleviate their psychological distress, the negative emotions that arise can destroy their self-understanding and attitudes toward life, leading to a loss of a sense of meaning in life (Huang et al., 2024).

In addition, all four groups of adolescents scored higher on meaning-seeking than meaning-possessing, which is consistent with the typical developmental pattern observed by Steger et al., 2008 of increases in meaning-seeking and decreases in meaning-possessing in adolescence (Lou, 2023). Previous studies have also found that in high school, adolescents’ level of meaning-seeking increases rapidly, and their level of meaning-possessing decreases rapidly (Qi et al., 2013), which may be because individuals show higher levels of self-differentiation and a more active search for meaning in life during high school (Xiang, 2021). However, some studies have highlighted that seeking a sense of meaning in life itself is not the main factor influencing individuals’ wellbeing; instead, the key aspect was whether the individual was able to become an active meaning-holder (Zhang et al., 2010). From this perspective, helping young people to explore the meaning of life so that they can truly have a sense of meaning should be a key focus of educational practice to promote the development of adolescents’ mental health.

### 4.3 Differences in social-emotional competence among adolescents with different perceived social support types

The finding of significant differences in social-emotional competence among adolescents in different perceived social support categories validates Hypothesis 3. Specifically, adolescents with rich levels of perceived social support exhibited the highest level of social-emotional competence (confirming Hypothesis 3a), whereas adolescents with poor levels of perceived social support showed the lowest level of social-emotional competence (confirming Hypothesis 3b). This result suggested that the higher the level of perceived social support, the greater the social-emotional competence of the adolescent (Qian and Cao, 2023). Interactive ritual chain theory proposes that individual interpersonal interactions in a given situation are driven by emotional energy (Deng, 2020). During adolescence, individuals’ multiple human–computer interactions with parents, peers, and teachers are a protective factor for social-emotional competence (Chen, 2023). Adolescents with poor perceived social support face chronic deprivation in multidimensional support resources, which is consistent with the cumulative risk model. Persistent ecological resource deficits limit their opportunities to engage in high-quality proximal interactions and impede the development of social-emotional competence (Zhou et al., 2016). Low levels of social-emotional competence, such as weak self-management skills and inadequate social awareness, significantly increase their risk of academic difficulties, behavioral problems, and psychological vulnerability, creating a vicious cycle.

Notably, peer-oriented adolescents did not perform as well as adolescents with a medium level of perceived social support in self-management and social awareness skills, which may be due to perceived teacher support during adolescence being significantly and positively correlated with students’ emotional intelligence (Zeng, 2022). Peer-oriented adolescents who perceive lower levels of teacher support may have deficits in self-emotional management and sensitivity to the emotional states of others. These factors are detrimental to the development of social-emotional competence and ultimately serve as key barriers to their academic engagement and campus adjustment. Thus, the supportive role of teachers in the development of adolescents’ social-emotional competence should not be overlooked and should be fully emphasized and actively promoted. More importantly, differences in social-emotional competence have far-reaching practical significance and are significantly related to adolescents’ academic performance, psychological resilience, and behavioral adaptation.

### 4.4 The mediating role of social-emotional competence

The present study further found that social-emotional competence played a mediating role between different perceived social support categories and sense of meaning in life, validating Hypothesis 4. Specifically, social-emotional competence played a stronger mediating role between the Rich type of perceived social support and sense of meaning in life—that is, social-emotional competence is a stronger predictor of interpersonal trust and is more effective at enhancing a sense of meaning in life when an individual perceives higher levels of social support (Yuan, 2024). Within the resource preservation perspective, the value-added spiral effect explains that individuals with resources are not only more capable of acquiring other resources, but that these acquired resources produce a greater incremental increase in resources, further enhancing social-emotional competence and a sense of meaning in life (Sun et al., 2024). Moreover, self-determination theory suggests that individuals tend to show self-integration and continuous adaptation under the support of appropriate external environments, and that social-emotional competence enhances intrinsic motivation and self-efficacy by satisfying the need for autonomy, competence, and a sense of belonging, which in turn enhances the sense of meaning in life (Zhang S. et al., 2022). Support from the external environmental usually involves an individual’s close social relationship network, and with the support of this network, the individual is able to continuously cultivate their social-emotional ability in different social situations, ultimately enhancing their sense of meaning in life. The results of this study also validate social support theory, which proposes that when individuals perceive more social support resources, they will be more inclined to establish close psychological ties with significant others, which in turn has a positive effect on the development of social-emotional competence (Li and Chen, 2023); thus, a high level of social-emotional competence significantly affects individuals’ search for and possession of a sense of meaning in life.

## 5 Conclusion

This study has revealed four categories of adolescents’ perceived social support: Poor, Rich, Peer-oriented, and Medium types, with the Rich and Medium categories being more commonly found within the sample. Differences were found in the sense of meaning of life across these categories, with adolescents in the Rich category having the strongest sense of meaning of life and those in Poor type the weakest. The study also identified differences in social-emotional competence between the different perceived types of social support, with adolescents within the Rich category having the strongest sense of meaning in life and those in the Poor category the weakest. Using the Poor type of perceived social support as the reference group, social-emotional competence was found to mediate the relationship between the Rich, Peer-oriented, and Medium types of perceived social support and sense of meaning in life.

### 5.1 Significance and limitations of the study

Theoretically, by revealing the four types of adolescents’ perceived social support, this study has innovatively explored the impact of the categories of perceived social support on the sense of meaning in life from the perspective of cumulative ecological resources. It has identified the types of perceived social support that are the most and least favorable to enhancing adolescents’ sense of meaning in life. This not only extends and supplements existing theories on perceived social support and the sense of meaning in life but also offers new theoretical perspectives for understanding the development of the sense of meaning in life.

In terms of educational practice, this study provides insights for improving the development of interventions for adolescents with poor and peer-oriented types of perceived social support. For adolescents in the poor category, efforts should focus on helping them to establish a comprehensive social support network and improve their overall level of perceived social support. For adolescents in the peer-oriented category, teachers should implement differentiated support strategies, while for individuals with a strong sense of autonomy, teachers should focus on providing instrumental support, such as learning to understand the meaning of through learning resources and strategy guidance. In contrast, for individuals with high sensitivity to emotional needs, teachers should provide more emotional support, including encouragement, affirmation, and care, in order to enhance these students’ sense of self-worth and belonging.

However, this study has certain limitations in its research methodology and data sources that should be addressed in future research. First, it relied primarily on adolescents’ self-reported data, which may introduce social desirability biases or common methodological biases that could affect the objectivity of the results. In addition, while the reliability and validity of the measures were confirmed, the self-reported nature of the measures cannot completely exclude individual comprehension or recall bias. Future research should adopt multi-source information validation strategies, such as parent, teacher, or peer ratings, and the construction of multi-subject reporting models to more comprehensively and objectively assess adolescents’ perceptions of social support and sense of meaning in life.

Second, caution needs to be taken regarding the representativeness of the study sample and the generalizability of the conclusions. The sample of this study was limited to parts of Shanxi Province, China, and the conclusions may be influenced by the cultural and socioeconomic context of this particular region. As mentioned in the discussion section, there are cultural differences in how social support is used. Therefore, whether the potential category patterns identified in this study and their effects on the sense of meaning in life can be generalized to other regions of China or even different cultural contexts needs to be further verified. Future research should expand the sample coverage and conduct cross-regional and cross-cultural studies to test the generalizability of the findings.

Third, there are fundamental limitations in the nature of the research design. This study utilized a cross-sectional design, and although it revealed significant associations between different social support categories and sense of meaning in life, it was not possible to determine a causal relationship between the two. For example, it was not possible to conclude whether it is the mode of support that influences the sense of meaning in life or whether adolescents with different levels of sense of meaning in life tend to perceive their social support in a particular way. In order to explore the mechanisms of their dynamic development in greater depth, there is an urgent need for future research to adopt a longitudinal tracing design that assesses these variables at different points in time, so as to reveal their causal paths and developmental trajectories more clearly.

Finally, there is still room to expand the scope of the research. For example, dissociative adolescents—who have unique psychological mechanisms and developmental needs—should be analyzed in depth. In addition, this study focused on the adolescent population, while the construction of meaning in life is a dynamic process that occurs throughout life. Future research could extend the scope to different developmental stages (e.g., early adulthood and middle age) to explore how the combinatorial model of perceived social support evolves with age and life tasks and has a sustained impact on an individual’s sense of meaning in life. This would provide a more relevant empirical basis for mental health education across different ages.

## Data Availability

The raw data supporting the conclusions of this article will be made available by the authors, without undue reservation.
